# BPH treatment: laser for everyone | *Opinion: YES*

**DOI:** 10.1590/S1677-5538.IBJU.2018.02.02

**Published:** 2018

**Authors:** Carlos A. R. Sacomani, Ricardo Vita Nunes

**Affiliations:** 1 Departamento de Urologia, AC Camargo Cancer Center, SP, Brasil; 2 Divisão de Urologia, Universidade de São Paulo, USP, São Paulo, SP, Brasil

**Keywords:** Lasers, Therapeutics, Prostatic Hyperplasia, Transurethral Resection of Prostate

During the past decades, transurethral resection of the prostate (TURP) has been the gold-standard procedure for surgical treatment of benign prostatic hyperplasia (BPH) and became the second most common surgery in men in the Western world (1). A number of other techniques were developed through the years, trying to replace TURP, including vaporization, microwave thermotherapy, transurethral needle ablation (TUNA) and various types of laser therapies. The rational of looking for new therapies for BPH lies on the intention of delivering the same results with less complications and adequate length of stay at the hospital or even as an outpatient procedure. Indeed, morbidity and mortality following TURP are continuous issues. Reich et al. (2) evaluated 10,654 patients that underwent TURP in state of Bavaria, Germany. The cumulative short-term morbidity rate was 11.1%. The most important complications were failure to void, surgical revision, bleeding, urinary tract infections and TURP syndrome. Rassweiler et al. (3) showed decreasing complication rates in a review conducted from 1989 to 2005. Bipolar TURP emerged as a significant evolution in the last years, especially because saline solution can avoid TURP syndrome and enables a greater volume of tissue resection However, Skolarikos et al. (4) recently showed similar results in safety and efficacy comparing monopolar and bipolar TURP, with the same possible complications throughout the years.

In the other hand, various techniques of lasers were compared to TURP. Currently, holmium laser (HoLEP) and Greenlight are two of the most common used. Greenlight was released in 2006 and improved from 80W to 180W output (5). The GOLIATH study (6) compared Greenlight (GL) 180W with TURP (Monopolar and Bipolar) and considered Gl non-inferior to TURP in terms of International Prostate Symptom Score (IPSS), Qmax and proportion of patients free of complications. However, early reinterventions were lower in the GL group. These data clearly show GL has the same efficacy as TURP in relieving the symptoms and obstruction, but with the advantage of less early postoperative problems (Clavien III complication) (7). Stone et al. (8) also described good results in patients with prostate size greater than 150mL (median 202mL). Performing TURP in such individuals remains challenging.

HoLEP is an enucleation technique and has the largest number randomized control trials available comparing TURP and open prostatectomy. A meta-analysis conducted recently demonstrated similar efficacy outcomes of bipolar TURP and photovaporization and better results than monopolar TURP (9). Gilling et al. (10) also described long-term results (mean 7.6 years). Although, HoLEP requires morcellation to retrieve the prostate tissue and needs a learning curve of 40 to 60 cases, it emerges as a novel widespread used procedure for surgical treatment of BPH. HoLEP success has been reproduced in a number of studies (11). Recently, some authors described similar results comparing HoLEP and 120W GL (12, 13).

All these studies focused on all patients with BPH. If we considered special populations such as those receiving anticoagulants and anti-platelet and therefore not with suitable indication for TURP, both HoLEP and GL appears as feasible procedures (14, 15). Rajih et al. (16) compared men with high medical risk (HMR) by the American Society of Anesthesiologists (class 3) with healthier individuals and demonstrated similar results according to IPSS, Qmax, postvoid residual volume and quality of life. HMR group had more readmissions (3.7% vs. 1.3%; p=0.04), but regarding the comorbidities of these patients, we can consider only a few number of patients (3.7%) needed a new hospitalization.

It seems the era of laser for BPH has finally come. Urologists must learn these new procedures and discuss the proper option with their patients.

## References

[1] Reich O, Gratzke C, Stief CG (2006). Techniques and long-term results of surgical procedures for BPH. Eur Urol.

[2] Reich O, Gratzke C, Bachmann A, Seitz M, Schlenker B, Hermanek P (2008). Morbidity, mortality and early outcome of transurethral resection of the prostate: a prospective multicenter evaluation of 10,654 patients. J Urol.

[3] Rassweiler J, Teber D, Kuntz R, Hofmann R (2006). Complications of transurethral resection of the prostate (TURP)-incidence, management, and prevention. Eur Urol.

[4] Skolarikos A, Rassweiler J, de la Rosette JJ, Alivizatos G, Scoffone C, Scarpa RM (2016). Safety and Efficacy of Bipolar Versus Monopolar Transurethral Resection of the Prostate in Patients with Large Prostates or Severe Lower Urinary Tract Symptoms: Post Hoc Analysis of a European Multicenter Randomized Controlled Trial. J Urol.

[5] Zhang X, Shen P, He Q, Yin X, Chen Z, Gui H (2016). Different lasers in the treatment of benign prostatic hyperplasia: a network meta-analysis. Sci Rep.

[6] Bachmann A, Tubaro A, Barber N, d'Ancona F, Muir G, Witzsch U (2015). A European multicenter randomized noninferiority trial comparing 180 W GreenLight XPS laser vaporization and transurethral resection of the prostate for the treatment of benign prostatic obstruction: 12-month results of the GOLIATH study. J Urol.

[7] Dindo D, Demartines N, Clavien PA (2004). Classification of surgical complications: a new proposal with evaluation in a cohort of 6336 patients and results of a survey. Ann Surg.

[8] Stone BV, Chughtai B, Forde JC, Tam AW, Lewicki P, Te AE (2016). Safety and Efficacy of GreenLight XPS Laser Vapoenucleation in Prostates Measuring Over 150mL. J Endourol.

[9] Cornu JN, Ahyai S, Bachmann A, de la Rosette J, Gilling P, Gratzke C (2015). A Systematic Review and Meta-analysis of Functional Outcomes and Complications Following Transurethral Procedures for Lower Urinary Tract Symptoms Resulting from Benign Prostatic Obstruction: Na Update. Eur Urol.

[10] Gilling PJ, Wilson LC, King CJ, Westenberg AM, Frampton CM, Fraundorfer MR (2012). Long-term results of a randomized trial comparing holmium laser enucleation of the prostate and transurethral resection of the prostate: results at 7 years. BJU Int.

[11] Kuebker JM, Miller NL (2017). Holmium Laser Enucleation of the Prostate: Patient Selection and Outcomes. Curr Urol Rep.

[12] Kim KS, Choi JB, Bae WJ, Kim SJ, Cho HJ, Hong SH (2016). Comparison of Photoselective Vaporization versus Holmium Laser Enucleation for Treatment of Benign Prostate Hyperplasia in a Small Prostate Volume. PLoS One.

[13] Cho MC, Song WH, Park J, Cho SY, Jeong H, Oh SJ (2018). Long-term outcomes of laser prostatectomy for storage symptoms: Comparison of serial 5-year follow-up data between 120W HPS photo-selective vaporization of the prostate and holmium laser enucleation of the prostate. J Urol.

[14] Rivera M, Krambeck A, Lingeman J (2017). Holmium Laser Enucleation of the Prostate in Patients Requiring Anticoagulation. Curr Urol Rep.

[15] Eken A, Soyupak B (2018). Safety and efficacy of photoselective vaporization of the prostate using the 180-W GreenLight XPS laser system in patients taking oral anticoagulants. J Int Med Res.

[16] Rajih E, Tholomier C, Hueber PA, Alenizi AM, Valdivieso R, Azizi M (2017). Evaluation of Surgical Outcomes with Photoselective GreenLight XPS Laser Vaporization of the Prostate in High Medical Risk Men with Benign Prostatic Enlargement: A Multicenter Study. J Endourol.

